# Feeding of *Agaricus bisporus* Mushrooms is Impacted by Mycelium Colonization Time and Network Architecture

**DOI:** 10.1111/1462-2920.70345

**Published:** 2026-06-15

**Authors:** Guus van Iersel, Johan Baars, Pieter Vervoort, Robert‐Jan Bleichrodt

**Affiliations:** ^1^ Microbiology, Department of Biology Utrecht University Utrecht the Netherlands; ^2^ CNC Grondstoffen Milsbeek the Netherlands

**Keywords:** *Agaricus bisporus*, fungal hydraulics, hyphal network architecture, mushroom cultivation, nutrient translocation, substrate depletion

## Abstract

Mycelial nutrient uptake and transport sustain mushroom growth, yet the physical factors constraining long‐distance translocation in *Agaricus bisporus* remain poorly understood. For instance, the bottom compost stratum is underutilized by the fungus to support its mushroom production. We investigated how mycelial network architecture and colonization time influence nutrient flow during cultivation. Using stable isotope labeling, we quantified mushroom tracer uptake from the lower compost stratum in distinct culture setups. The total resource uptake by a mushroom is determined by the flow of resources to that mushroom and is affected by water and nutrient uptake by the vegetative mycelium in compost. Hydraulic resistance across the network limits this resource flow and consequently limits mushroom growth. We found that increasing cord abundance can counteract this limitation and enhance productivity when long‐distance translocation is required due to local nutrient depletion. Extended colonization time increases total resource uptake, as longer growth periods enhance network density and substrate exploitation. We found that mushrooms within a flush heterogeneously feed from different compost strata, suggesting that fruiting bodies are heterogeneously connected to the mycelial network. For commercial cultivation, the findings highlight the importance of thorough substrate degradation and sufficient flow speed, achievable through extended colonization and cultivation.

## Graphical abstract

Resource flow through the mycelium follows Poiseuille's law: pressure differences between vegetative and fruiting body mycelium (∆*Ψ*), shaped by compost quality, colonization time, and local depletion, drive transport, while the number, radius, and length of hyphae (*n*, *r* & *L*) determine hydraulic resistance, acting against resource flow to developing mushrooms.

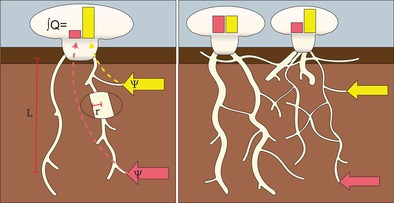

## 1 Introduction

Edible mushrooms, including *Agaricus bisporus*, account for a significant proportion of global agricultural output, with worldwide annual production estimated at around 43 million metric tons in 2018–2019 (Singh et al. 2020). Mushrooms have high nutritional value, are rich in protein, fiber, and essential nutrients, and have a lower environmental footprint compared with animal‐based foods, supporting their role as a sustainable dietary choice (Goglio et al. 2024; Robinson et al. 2019; Valverde et al. 2015). The well‐established cultivation practices of *A. bisporus* make it an ideal model organism for advancing sustainable mushroom production.

The cultivation of *A. bisporus* typically begins with the preparation of a nutrient‐rich composted substrate. In the Netherlands, this compost comprises wheat straw, horse/chicken manure, and gypsum which are derived from raw materials and waste streams originating from agriculture and the building industry (Gerrits 1988). Compost first undergoes partial microbial decomposition (Phase I; PI), followed by pasteurization and conditioning driven by thermophilic microbes (Phase II; PII). The substrate is then inoculated with grain spawn, initiating the spawn‐run phase (Phase III; PIII) and the fungus is allowed to colonize the compost in 16–19 days. Next, a casing layer is added (Noble et al. 2009) (Phase IV; PIV) that is colonized from the mycelium within the compost and by mixing in a small amount of PIII compost, a process known as compost added casing (CACing; (MacCanna and Flanagan 1972)). During casing colonization, temperature and CO_2_ are higher, but to induce fructification, temperature and CO_2_ levels are reduced by venting (Baars et al. 2020; Eastwood et al. 2013). *A. bisporus* produces its mushrooms during multiple harvests, or “flushes,” over several weeks. Commercially, two flushes are usually harvested before production and quality start declining, making it more economical to start a new production cycle.

*A. bisporus* compost production and disposal of spent substrate represent major economic and environmental costs (Goglio et al. 2024; Robinson et al. 2019). However, spent mushroom compost, mushroom stipes and mishappen mushrooms are being reused to produce other mushroom crops or mycelium materials (Dedousi et al. 2024; Zhao et al. 2025). A key issue is the uneven utilization of the substrate by *A. bisporus*, with the bottom layer being less effectively used compared with the top layer near the developing fruiting bodies (Sonnenberg et al. 2022; Vîta et al. 2026). During cultivation, ~70%–75% of the original carbohydrates and 40%–45% of the lignin are degraded or modified, leaving a significant part unutilized after the second flush (Jurak et al. 2014, 2015; Kabel et al. 2017). This underutilization limits the overall cropping efficiency and increases waste. While reducing the substrate depth can improve the biological efficiency of the cultivation, it also decreases the total yield of the mushroom bed, as the available substrate volume directly affects production potential and mushroom quality (Sonnenberg et al. 2022). In existing agricultural practices, this trade‐off is not favourable for farmers, as maximizing productivity remains a priority over improving substrate efficiency. Enhancing nutrient extraction by the fungus, from especially the bottom layer, is therefore crucial for making mushroom cultivation more sustainable and economical.

Our hypothesis is that mycelium architecture and colonization time within the compost affect nutrient extraction from different layers to feed the developing mushrooms. Mycelium architecture refers to the way the fungus colonizes the substrate, including the density of hyphal growth and the extent to which hyphae aggregate into cords. Fine, dense mycelium and cord‐like structures each offer distinct advantages in nutrient dynamics. Fine mycelium, with its extensive surface area, is better suited for effective substrate breakdown and nutrient uptake, allowing for localized extraction of resources (Tlalka et al. 2008). By contrast, cords formed by the aggregation of multiple hyphae facilitate efficient long‐distance nutrient transport within the mycelial network, redistributing resources across different substrate regions (Heaton et al. 2010; Herman et al. 2020; Herman and Bleichrodt 2021). From a physical perspective, this shift from diffuse to aggregated growth reduces hydraulic resistance, thereby facilitating long‐distance mass flow. In natural ecosystems, fungi utilize cords to be able to exploit specific ecological niches, such as decomposing organic matter or colonizing nutrient‐poor environments (Aguilar‐Trigueros et al. 2022; Fricker et al. 2008). The ability of fungi to adapt their growth forms and nutrient acquisition strategies to varying conditions is essential for their survival and competitive success (Heaton et al. 2010). Understanding these architectural strategies in an ecological context provides insights that can help optimize nutrient dynamics in controlled cultivation settings, even if the specific species being studied, such as *A. bisporus*, is grown outside its natural habitat.

The time that the mycelium is allowed to colonize its substrate plays a crucial role in these dynamics. Older mycelium typically has formed a denser network of hyphae, thus resulting in more thorough colonization of the substrate. This increased hyphal density enhances the mycelium's capacity for substrate degradation, nutrient absorption and transport, supporting fruiting body development. On the other hand, older mycelium may start to deteriorate, impacting these functions. We hypothesize that colonization with both fine mycelium and cord structures is essential for optimal nutrient uptake and transport, respectively, ensuring optimal feeding of mushrooms across multiple flushes. The exact balance between fine mycelium and cord abundance may shift during cultivation, depending on the demand of the developing mushrooms.

Nitrogen is a crucial nutrient in *A. bisporus* cultivation, yet it is relatively scarce in the substrate, which contains ~2% nitrogen by weight on a dry basis (PII compost) (Noble and Gaze 1996; Fidanza et al. 2010). This limited availability can restrict mycelium growth and fruiting body development, making efficient nitrogen utilization essential for maximizing yield. To overcome this limitation, growers often supplement the substrate after colonization with complex nitrogen‐rich additives, such as soybean meal or cereal brans. These supplements are commonly denatured to ensure slow release of nitrogen. They have been shown to significantly enhance productivity by providing an additional nutrient source that supports sustained growth and increased mushroom output (Carrasco et al. 2018). In previous research, we showed that nitrogen accumulation in mushrooms increases during successive flushes. For instance, the second flush relies more on protein and free amino acid for its fruiting body growth (van Iersel et al. 2025). In this study, we therefore focused on nitrogen transport.

To address the challenge of suboptimal substrate utilization in *A. bisporus* cultivation, this research focused on the question: *How do colonization time and mycelium network architecture influence nutrient uptake from the bottom layer of the substrate and feeding of the mushrooms?*

To investigate this, we employed a combination of a stable isotope labelling approach using ^15^N ammonium and dry matter content analysis during the cropping process. These methods allowed us to trace ^15^N and general nutrient uptake and long‐distance transport within the mycelium and towards the mushrooms. We found that network architecture and colonization time had marked effects on tracer transport to the mushrooms, depending on the conditions. This knowledge can inform strategies for more optimal and thereby sustainable *A. bisporus* cultivation.

## 2 Materials and Methods

### 2.1 Growth Conditions and ^15^N Labelling

**FIGURE 1:**
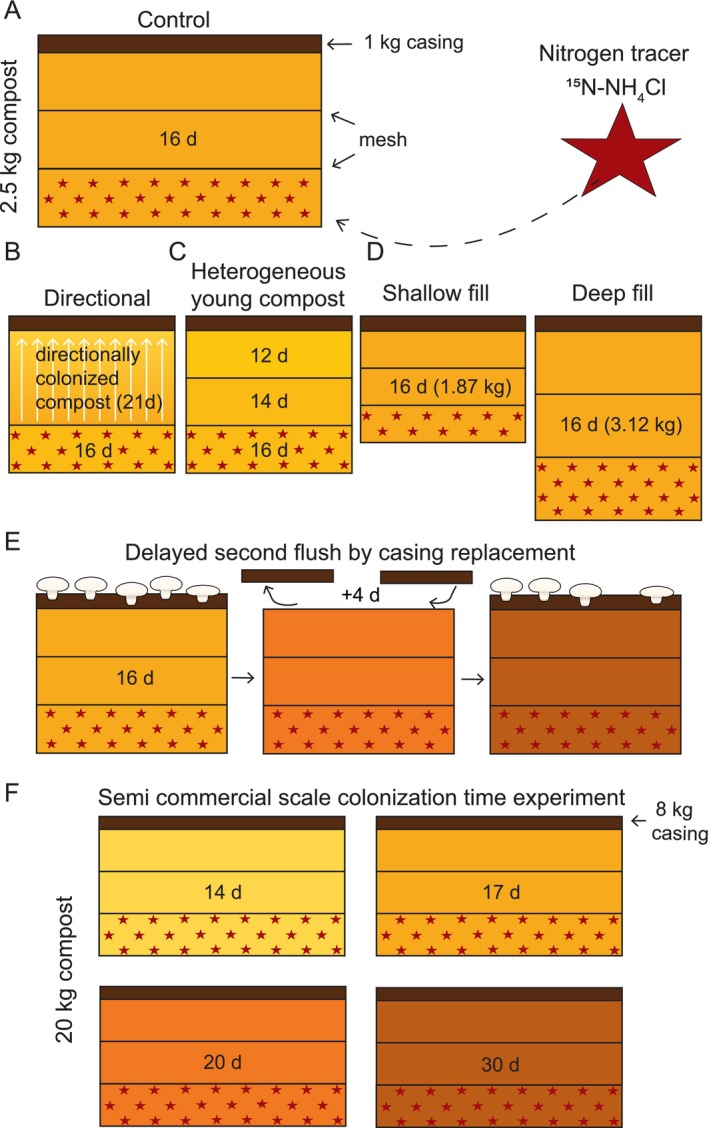
Overview of the various experimental setups used in this study. (A) Control setup using 16 days colonized compost and ^15^N tracer added to the bottom layer. (B) Directional growth experiment was set up using a pre‐grown block of directionally colonized PII compost placed on top of 16 days colonized compost with ^15^N tracer. (C) Heterogeneous colonization time experiment, top and middle layers were colonized for 12 and 14 days, respectively, before casing was added. (D) Filling depth experiment, 16 days colonized compost was filled either at control depth (~22 cm), 25% thinner (~16 cm) and 25% thicker (~26 cm), label concentration was kept constant. (E) In the delayed 2nd flush experiment, casing was removed after harvesting flush one, left uncovered for 4 days after which new casing was added and allowed to colonize under vegetative growth conditions. Then, cultures were placed back at fruiting inducing conditions. (F) Semicommercial colonization time experiment. For each treatment the compost had been colonized for a distinct time (i.e., 14, 17, 20 or 30 days). In cases where only the colonization time is indicated in the middle compost layer, also the top and bottom compost layer were of the same colonization time. If no compost weight is indicated, it is standard 2.5 kg (B‐E).

For all experiments, *A. bisporus* strain A15 (Sylvan, Horst, the Netherlands) was used. Phase 2 (PII) and Phase 3 (PIII) compost (16–19 days old) were supplied by CNC Grondstoffen (Milsbeek, the Netherlands) and casing soil by Legro (Katwijk, the Netherlands), with fresh batches provided for each experiment. Lab scale cultivation took place in 30 × 22 × 20 cm boxes (Euronorm, Manutan) with 5 holes of 1 cm diameter in the bottom for drainage, in triplicat. In all lab scale experiments, the bottom layer consisted of 833 g of PIII compost, labelled with 0.140 g ^15^N‐NH_4_Cl in 10 mL Milli‐Q water, added in drops spread across the surface and mixed thoroughly by hand. The top two layers, each comprising 833 g of PIII compost, were left unlabeled, but were modified depending on the experimental conditions (see Figure 1). Each box was topped with 1 kg of casing soil mixed with 20 g PIII compost (CACed).

Colonization of the substrate and casing occurred at 22°C and 85% relative humidity. After the casing was colonized, the boxes were transferred to venting conditions at 18°C. Watering was done four times over 72 h with 150 mL each watering after adding the casing and three times with 125 mL each after the first flush, spread out across 2 days. In case we observed excessive drying out of the casing soil, an additional 125 mL water was given to all boxes. Two flushes were harvested from all cultivations and subsequently analysed by Isotope Ratio Mass Spectrometry (IRMS).

### 2.2 Non‐Directional Growth

The bottom layer consisted of 833 g of ^15^N‐labelled PIII compost inoculated with spawn mixed throughout. The top two layers each comprised 833 g unlabeled PIII compost (Figure 1A).

### 2.3 Directional Growth and Heterogenous Young Compost

Directional cultures were inoculated using unlabeled 16‐days‐old PIII compost as inoculum (833 g) placed at the bottom and topped with 1666 g PII compost, to allow for colonization of the PII compost layer in a directional manner like described by Herman et al. (2020). PIII and PII compost were separated by a layer of fibreglass mesh, to allow their separation later on. After 21 days, the directionally colonized block (previously PII compost) was placed on a layer of the same batch of 16 days old PIII compost with ^15^N‐label (Figure 1B). Additionally, a test was done with 2 colonization times of compost to mimic the heterogeneity in colonization time between different layers of directional growth. To this end, 12 days old and 14 days old non‐directionally grown composts were used as the top and middle layer, respectively, which correspond to the average age of the directional layers (Figure 1C). The bottom layer comprised 16 days old ^15^N‐labelled PIII compost.

### 2.4 Varying the Filling Depth

To investigate how filling depth influences the utilization of compost strata, three different filling depths were tested. The control treatment consisted of 2.5 kg of compost topped with casing, resulting in a total bed depth of ~22 cm (Figure 1A). A shallow fill treatment contained 1.87 kg of compost (~16 cm), while the deep fill treatment contained 3.12 kg (~26 cm) (Figure 1D). For the deep fill treatment, the capacity of the standard Euronorm boxes was increased by attaching a plexiglass extension rim (6 cm height), allowing for the additional compost volume. The bottom compost layer was labelled with ^15^N‐ammonium chloride, with the absolute volume of labelled compost varying relative to the total compost volume, amounting to 1/3 of the total compost (Figure 1D).

### 2.5 Delaying the Second Flush by Replacing the Casing Layer

To delay the second flush and study how this affects resource uptake, a setup similar to the control setup was used (Figure 1E). After the first flush, the casing layer from 3 boxes was removed, and de‐cased boxes were held under vegetative growth promoting conditions for 4 days, after which new casing was added. Casing was CACed with minor amounts of compost from the cultivation itself (no new compost was added). After colonization of the casing (7 days), boxes with newly colonized casing were transferred to fructification inducing conditions again (Figure 1E). Delaying fructification leads to a total time of 18 days between the first and the second flush, compared with 7 days when casing is not replaced (control *n* = 3). Mushrooms from both the control and boxes with delayed second flush were harvested and analysed for ^15^N accumulation using IRMS.

### 2.6 Analysing ^15^N Accumulation Within the Substrate and Mushrooms During Development

The control setup with 3 layers of 16 days colonized PIII compost was used (Figure 1A). Coated fibreglass mesh was used to separate the compost layers. After pinning, the first flush and the second flush, 3 replicates were sampled from the casing layer and each of the substrate layers (top, middle, and bottom). Layers were weighed and homogenized, and samples of 60–100 g were taken, freeze dried, and ground using a pulverizing mill (Herzog, HP‐MA) for ^15^N enrichment analysis (EA‐IRMS).

Using the control setup (Figure 1A), fruiting bodies were harvested at five developmental stages. Pins were small and spherical (mean mass 0.4 g). Buttons were harvested 2 days later when cap and stipe were similar in size (mean mass 6 g). Small mushrooms were harvested 2 days later, 1 day before commercial size (mean mass 33 g). Commercial‐stage mushrooms were harvested when the veil was still intact (mean mass 76 g). For the open‐cap stage, three fruiting bodies were left to mature for four additional days after reaching commercial size (mean mass 198 g). At each developmental stage, three fruiting bodies were harvested and pooled for analysis.

### 2.7 Analysis of ^15^N Labelling Heterogeneity Between Individual Mushrooms

Seven individual mushrooms were picked from control boxes (Figure 1A) with the bottom compost layer being labelled with ^15^N‐ammonium chloride (Figure 1A), during 3 separate experimental trials during the first flush. Cultivation boxes were photographed, and sampled mushrooms were numbered. Mushrooms were dried, ground, and analysed for ^15^N and total N accumulation using EA‐IRMS. Dry matter content was determined and protein content was measured using a BCA assay kit (Thermo Fisher Scientific), with bovine serum albumin (BSA) as the standard. Technical duplicates of each biological replicate were performed and the mean of the two was reported (mean CV 4.8%). Amino acid content was determined as previously described (van Iersel et al. 2025). Osmotic potential (MPa) was measured using an osmometer (Vapro^tm^ 5520, Wescor, Logan UT, USA).

### 2.8 Semi‐Commercial Compost Colonization Time Experiment and Feeding of Mushrooms

To study the effect of compost colonization time on substrate utilization across strata, one batch of PII compost was used and sufficient compost was flash cooled and stored until needed for inoculation. 180 kg batches of PII compost were taken out from cooling 2 days before inoculation to reach room temperature (RT). 14, 17, 20, and 30 days prior to filling, batches of RT PII compost were inoculated with 5 g/kg A15 spawn (Sylvan, Horst, the Netherlands). Spawn was mixed thoroughly using a pitchfork on a large pile and divided into 22 kg portions and filled into boxes (inside dimensions 56 × 36 cm) and covered with plastic to prevent evaporation. During colonization, temperature was monitored and adjusted so that the internal temperature did not exceed 30°C. At the day of filling, compost contents of boxes were combined per colonization time group and mixed into a pile, after which new boxes were filled into 3 layers of 6.67 kg compost each, separated by plastic mesh. The bottom layer was labelled with 0.56 g ^15^N‐ammonium chloride dissolved in 60 mL dH_2_O, and mixed for 1 min by hand to homogenize. Samples were taken from each compost batch and analysed for dry matter content. Labelled layers were also sampled for ^15^N content determination. Compost was topped with 8 kg casing soil mixed with 250 g of the corresponding colonized compost to simulate CACing (Figure 1F). Boxes were further treated alongside commercial cultivations (Maatschap van den Heuvel, De Rips, the Netherlands). Harvesting of 2 successive flushes took place noting daily production. Of the 8 boxes per colonization time of compost, 4 were destructively harvested after the first flush. Layer wet and dry weight was measured to determine the dry matter content of the compost. The bottom compost layer was also analysed for remaining ^15^N tracer. After the second flush, the remaining 4 boxes were analysed in a similar manner. Mushrooms produced during the first flush from all 8 boxes and the 4 remaining boxes remaining during the second flush were analysed for ^15^N content. For each box, 10 individual mushrooms were randomly selected, quartered, pooled, and sampled.

### 2.9 EA‐IRMS, Sample Preparation and ^15^N and Total Nitrogen Quantification

Samples consisted of 10 mushrooms randomly picked from across the cultivation box surface. Per box, mushrooms were pooled except for the heterogeneity analysis. The feet of stalks of picked mushrooms were cut and discarded, as is routinely done in commercial mushroom cultivation. Mushrooms were longitudinally quartered; subsequently, quarters were pooled and freeze‐dried. The dried tissue was ground into powder using 10 ball bearings (3/16″) in 50 mL Greiner tubes shaken for 9 min at 11.33 Hz in a paint shaker (SK550, Fast & Fluid). Total nitrogen and nitrogen isotope composition (^15^N/^14^N) in ground samples was determined using Elemental Analyser—Isotope Ratio Mass Spectrometry (EA‐IRMS). To this end, ~2000 μg samples were weighed and combusted in tin capsules (5 × 9 mm; Elemental Microanalysis, UK). The following ~200 μg standards were used: Ammonium sulphate standard (Merck) and nicotinamide (Merck). Standards and samples were then measured using a Flash IRMS elemental analyser (Thermo Scientific) coupled to a Delta V Advantage IRMS (Thermo Scientific).

### 2.10 Statistical Analysis

Statistical analyses were performed separately for each flush and, where relevant, on treatment totals. Multiple means were compared using an ANOVA with Tukey HSD post hoc test. In case the ANOVA test was not significant this is indicated with NS in figures. Significantly different effects from the post hoc test are indicated with different letters of specific colour for distinct comparisons. Pairwise comparisons between two treatments were analysed using Student's *t*‐test and are indicated with brackets in figures. NS = not significant and otherwise *p*‐values are indicated. Differences were regarded as significant when *p* < 0.05.

## 3 Results

### 3.1 Individual Mushrooms Obtain Nitrogen From Different Substrate Layers

**FIGURE 2:**
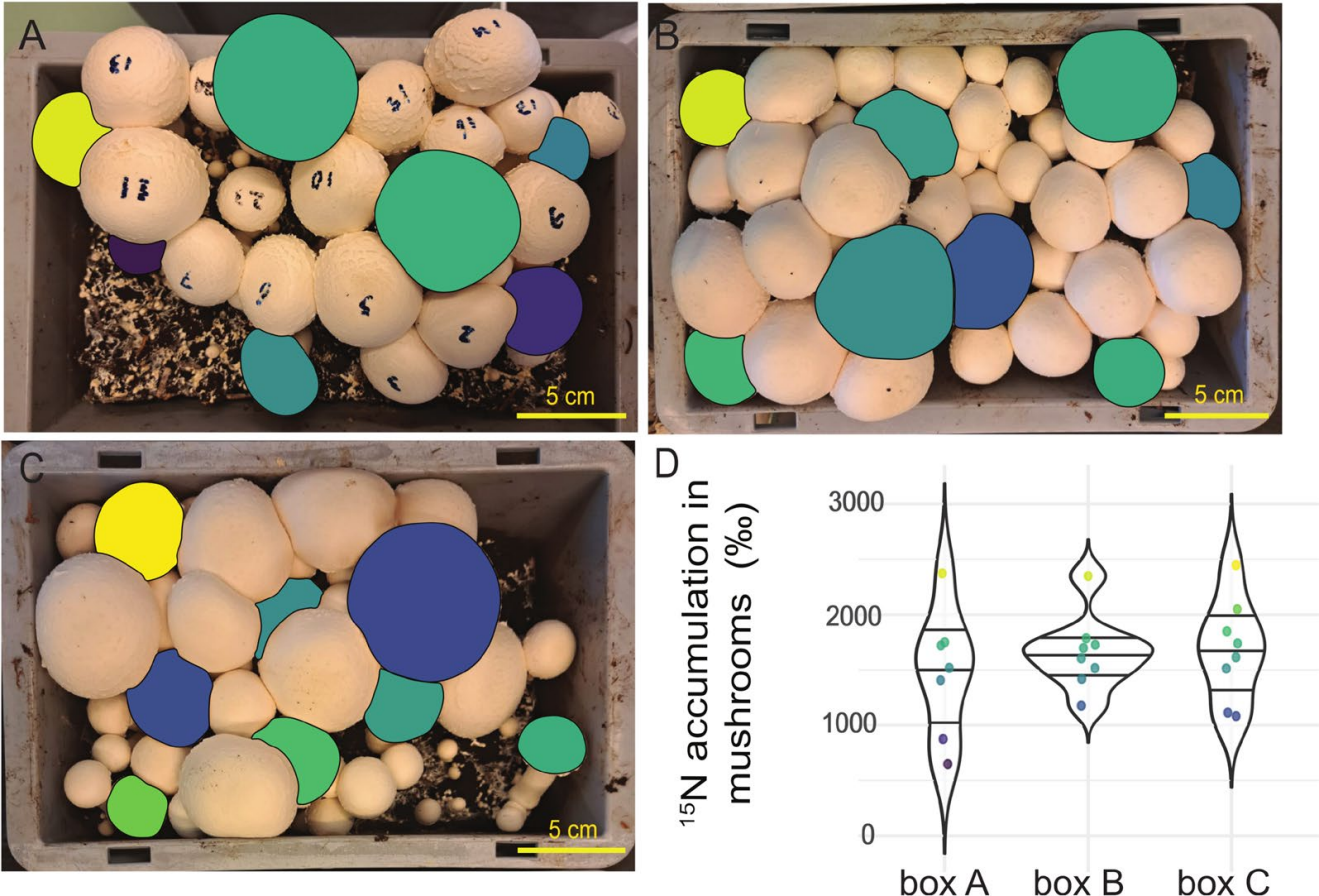
Heterogeneous ^15^N incorporation by mushrooms on the same mushroom bed. (A‐C) Three boxes of control setup cultivations (Figure 1A) during 3 separate cultivation cycles. During flush one, 7–8 individual mushrooms were harvested per box. ^15^N accumulation within mushrooms on the bed is indicated using a colour gradient (see panel D for colour legend). A strong degree of heterogeneity exists between individual mushrooms in a box culture with ^15^N‐labelled bottom layer. Even mushrooms topographically close to one another show drastically different uptake of ^15^N from the bottom layer (A‐C). However, averaged total uptake from different layers is comparable between boxes (D).

To examine how nitrogen is allocated to individual fruiting bodies within a shared mycelial network, ^15^N‐labelled nitrogen was applied to the bottom compost layer and tracer incorporation was measured in individual mushrooms (Figure 1A). Individual mushrooms varied strongly in their source of nitrogen. Pins initiated throughout the bed, but only a subset developed into mature fruiting bodies. Among those harvested, ^15^N incorporation from the bottom layer differed by more than six‐fold between individual mushrooms (Figure 2). No consistent relation was observed between tracer enrichment and the position of the fruiting body. Similarly, mushroom size, dry matter content, osmotic potential, amino acid and protein content showed no clear correlation with tracer uptake. Only total nitrogen concentration showed a weak but significant positive correlation (*R* ^2^ = 0.30) (Tables S1 and S2). This shows that mushrooms within the same flush feed from different compost layers, even though the network has a high degree of interconnectivity.

Substantial variation in tracer incorporation was observed among mushrooms harvested from the same box (δ^15^N 1608‰ ± 450‰; CV = 28.0%). Since treatments were applied at the level of the cultivation box and the mycelium inside each box can be seen as a biological unit. Each box culture was considered the experimental unit in subsequent experiments. Individual mushrooms were therefore treated as subsamples of the same unit. Pooling multiple mushrooms provides an estimate of the mean tracer incorporation in fruiting bodies by a single culture. Based on the observed variance among individual mushrooms, pooling 10 mushrooms per box would reduce the expected sampling error of the mean by approximately √10, resulting in an estimated standard error of 142.3. This sampling uncertainty is smaller than the observed treatment differences reported in the following experiments. This indicates that the observations made on pooled mushroom samples reflect genuine biological effects.

### 3.2 The Mycelial Network Architecture Impacts Feeding of the Mushrooms

The bottom layer of the compost is underutilized for feeding the developing mushrooms (Sonnenberg et al. 2022; Vîta et al. 2026). Therefore, we tested if cords that have the potential of serving as highways for nutrient transport (Herman et al. 2020) enhance long‐distance nutrient translocation to mushrooms. To this end, we compared directionally grown fungal networks (many cords) to industry standard homogeneously non‐directionally colonized compost (fine mycelium, few cords). For directional growth, we replaced the top two layers of the substrate with a block of compost that was pre‐colonized in a directional manner (Figure 1B). For the homogeneously colonized compost control, 16 days colonized compost was used that was homogenized (Figure 1A).

**FIGURE 3:**
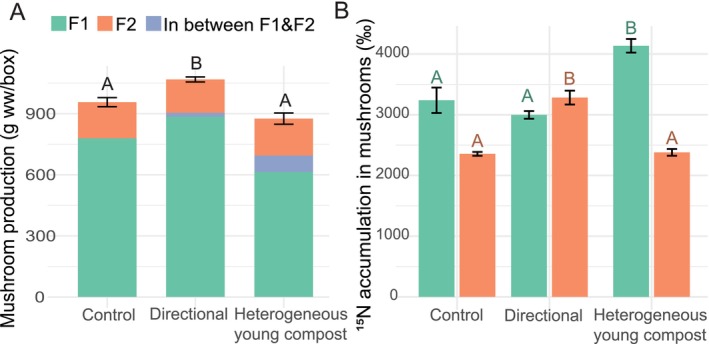
(A) Total yield (ww = wet weight) is significantly increased during directional growth across 2 flushes, mainly due to increased yield in the first flush. Heterogeneous young compost had reduced yield, mainly in the first flush. First flush (green bars), Second flush (orange bars), In between flushes (blue bars). (B) ^15^N accumulation in the first flush (green bars) and the second flush (orange bars). The control shows a significant decrease of bottom layer ^15^N utilization in the second flush. Directional growth uses ^15^N from the bottom layer more during the second flush compared with the control. Heterogeneous young compost has a similar pattern compared with the control, but is more extreme, and with more bottom layer ^15^N utilization in the first flush. Bars show means ± SE. Different letters indicate significant differences (Tukey HSD). Differently coloured letters indicate comparisons either between flush 1 or between flush 2.

The timing of both the first and second flush did not differ between the directionally grown cultures and the control. However, directionally grown cultures produced 11.7% more yield (*p* = 0.025), mainly resulting from a larger first flush compared to the control, while the second flush was similar in both groups (Figure 3A).

Contrary to our hypothesis, the mushrooms of the first flush did not exhibit a significantly different ^15^N tracer uptake from the bottom layer in directional growth cultures compared with the control. Despite higher yields in the directional cultures, the total absolute ^15^N nitrogen uptake (yield × ^15^N enrichment) from the bottom layer was only slightly increased due to the higher yield, despite the slightly lower ^15^N concentration of the mushrooms (Figure 3A,B).

In the second flush, under control conditions, the developing fruiting bodies took up 27.3% less ^15^N from the labelled bottom layer compared with the first flush. In contrast, with directional growth conditions, ^15^N uptake from the bottom layer increased by 9.5% (Figure 3B). After 2 flushes, this meant that directional cultivation, besides the increased yield, also used 14.5% more ^15^N from the bottom layer (*p* = 0.13).

Directional cultivation does not only result in abundant cord formation, but also creates a mycelium of heterogeneous colonization time, in which the mycelium close to the inoculum is oldest and the mycelium close to the casing is youngest. This heterogeneous colonization time could impact nitrogen utilization by the mushrooms. Therefore, we tested the impact of heterogeneous colonization time on ^15^N transport. The total block of directional growth used needed 21 days for full colonization from bottom to top. This meant that the top layer had a relatively short colonization time compared to the middle and bottom layer. Accordingly, we mimicked this heterogeneous colonization time between layers in directionally grown cultures, but with less cord formation. To this end, the top layer and middle layer were filled with 12 days old and 14 days old homogeneously colonized compost, respectively (Figure 1C), while the bottom layer was 16 days old in both treatments. In the first flush, the bottom layer supplied relatively a lot of resources, resulting in the uptake of 27.7% more ^15^N tracer (Figure 3B), however, this could not sustain the same yield as observed in the control (−21.3%) (Figure 3A). By the onset of the second flush, the younger top layers had more time to break down the substrate, thereby supplying more nutrients to the growing mushrooms, and as a result tracer uptake became similar to the control (Figure 3B). Thus, the young heterogeneous compost is less capable of supplying nutrients from the top compost layer in flush 1. However, the young mycelium in the top layer does facilitate sufficient translocation of nutrients derived from the bottom layer during the first flush.

### 3.3 Compost Colonization Time

To further test how compost colonization time influences substrate utilization by the mushrooms, a semi‐commercial trial was conducted with PII compost colonized for 14, 17, 20, or 30 days. The bottom layer was labelled with ^15^N–NH_4_Cl. These cultures were run in parallel with a commercial cultivation cycle; therefore, time before venting was adjusted to that specific commercial cultivation. Nevertheless, flushes occurred more or less simultaneously for all treatments. Two flushes were harvested for analysis of yield, ^15^N accumulation, and the substrate dry weight change (Figure 1F).

**FIGURE 4:**
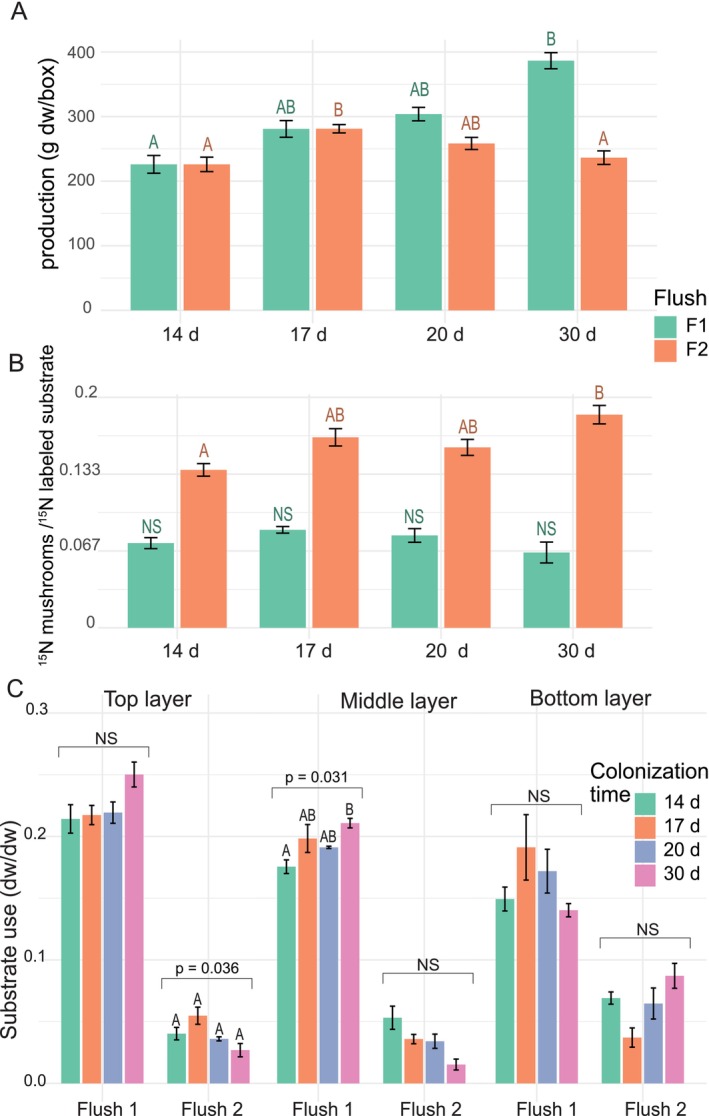
Effect of mycelium colonization time in compost on yield and layer utilization. Compost was colonized for 14, 17, 20 or 30 days before filling into 3 layers separated by netting in boxes (3 × 6.67 kg) with the bottom layer ^15^N‐labelled. (A) Average yield (dw = dry weight) per box separated by flush (F1 = flush 1 = green bars, F2 = flush 2 = orange bars). Fourteen day compost produced the lowest total yield, while 30 days colonized compost produced the highest yield, mainly due to differences in flush 1. (B) ^15^N incorporation into fruiting bodies, reflecting bottom‐layer ^15^N use. In all colonization times, incorporation was higher in flush 2 than in flush 1, with the largest increase in 30 days colonized compost. Different letters indicate significant differences (Tukey HSD); NS = not significant (ANOVA). (C) Dry matter loss per layer (dw/dw (kg/kg)) across flushes: 14 days (green), 17 days (orange), 20 days (blue), or 30 days (pink). Bottom layers tended to lose more weight during flush 2, though variability was high. Overall high use of a layer in F1 seemed to lead to low F2 use in that same layer and vice versa. Bars show means ± SE. Brackets (C) show one‐way ANOVA comparison of colonization times within a specific layer and flush, NS = not significant. Different letters indicate significant differences (Tukey HSD). (A, B) Differently coloured letters indicate comparisons either between flush 1 or between flush 2.

Compost colonization time significantly affected yield (Figure 4A). Fourteen‐day colonized compost gave the lowest production (248 + −13 g kg^−1^ compost), mainly due to poor flush‐one performance. Thirty‐day colonized compost yielded significantly more than all other colonization times (336 + −27 g kg^−1^ compost), with the difference expressed primarily in flush one (Figure 4A). Seventeen and twenty‐day colonized compost were intermediate and statistically similar.

Across all colonization times, most compost dry matter loss (between 17.1% and 21.3%) occurred before and during the first flush. This dry matter was only partially transferred to mushroom dry matter (between 2.7% and 6.6%). In flush two, most of the dry matter loss was explained by dry matter gain in the mushrooms (Figure 4C). Thus, respiration played a larger role on the mass balance during the first flush. The top layer was most strongly exploited in the first flush ~21% reduction across shorter colonization times and 25% in 30 days colonized compost, this led to very low utilization in the second flush (Figure 4C). The middle layer showed the same pattern, with a significant colonization time effect in flush one, highest loss in 30 days, lowest in 14 days colonized compost. In the second flush, middle layer dry weight reduction was limited akin to the top layer. The bottom layer was less used than the top and middle compost layer in flush one (14%–19% reduction), but became a more important source during flush two (4.1%–5.3% reduction), compared with the top and middle compost layer (Figure 4C). Differences between colonization times within the same layer and timepoint were mostly not significant due to high variability, though a reciprocal trend between flushes was apparent (Figure 4C). Mushrooms in treatments that did use the bottom compost layer more in flush one did less than mushrooms in flush two, and vice versa.

Tracer data provided a clearer signal of colonization time effects. In the first flush, no significant difference in ^15^N uptake was observed (Figure 4B). In the second flush, ^15^N uptake showed that older composts, especially 30 days colonized, mobilized significantly more ^15^N from the bottom layer than 14 days colonized composts, both in relative terms (32% more (δ^15^N)), but also in absolute terms (38% more (g ^15^N)) (Figure 4B). This is reflected in the dry weight analysis (Figure 4C), but there it is less clear as variability masked colonization time differences. The discrepancy likely reflects that dry weight loss also includes respiration, casing colonization, and leaching, while tracer uptake directly tracks ^15^N incorporation into mushrooms, since it is not lost by respiration.

### 3.4 Delaying the Second Flush

**FIGURE 5:**
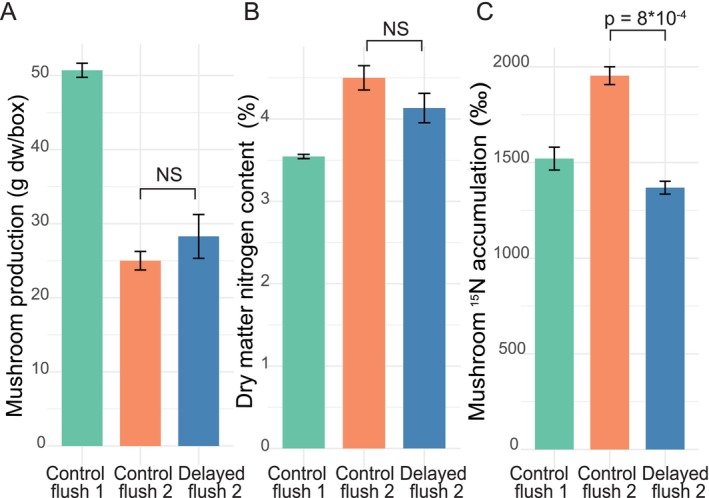
(A) Delaying the second flush by 11 days through casing replacement results in a slight non‐significant increase in second flush productivity. Flush 1 (green bars), Control Flush 2 (orange bars), Delayed flush 2 (blue bars). (B) Nitrogen content (dw/dw) of mushrooms. (C) ^15^N content in mushrooms increases during the second flush in the control; this increase is absent when the 2nd flush is delayed. A significant reduction in long‐distance ^15^N translocation is observed during the delayed second flush. Data represent mean ± SE. *p*‐values from Student's *t*‐tests indicate significance between the control and delayed second flush indicated above brackets, NS = not significant.

We hypothesized that the time between flush 1 and 2 is too short to give the mycelium sufficient time to further degrade the substrate to be able to optimally feed the second flush of mushrooms. To test this, the casing layer was removed after the first flush and de‐cased cultures were held at vegetative growth conditions for 4 days, after which new casing was applied and further incubated for 7 days. Thereafter, cultures were placed at mushroom inducing conditions again. This delayed the second flush by 11 days (18 days vs. 7 days between flushes in the treatment vs. the control). Yield in the second flush increased from 25.0 ± 1.5 to 28.3 ± 3.0 g dry weight (dw) per box, but this difference was not significant (*p* = 0.39) (Figure 5A). Strikingly, the delayed second flush did not show the usual increase in bottom‐layer exploitation: ^15^N uptake from the bottom layer remained at first‐flush levels, and was significantly lower than that of the second flush of the control (p = 0.0008), indicating an even more uneven substrate use between the layers when the flush was delayed. Consistently, the nitrogen content of mushrooms, which normally rises in the second flush (van Iersel et al. 2025), also increased less (16.5%) than in the control (26.9%); however, this was not significant (*p* = 0.19) (Figure 5B).

### 3.5 Influence of Compost Depth on Bottom‐Layer ^15^N Uptake

**FIGURE 6:**
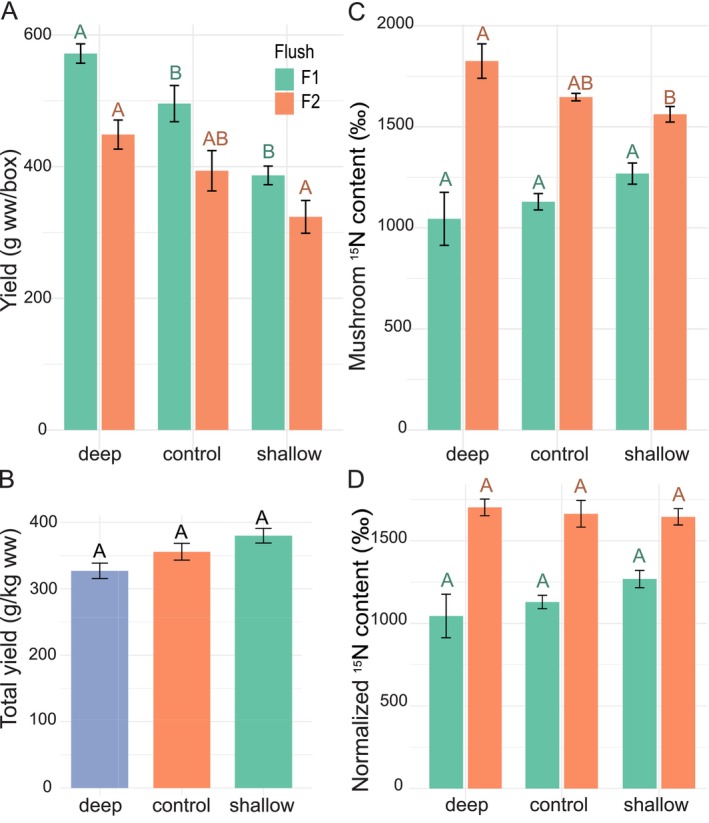
Bottom‐layer utilization across filling depths and flushes. Boxes were filled with shallow (16 cm), standard (22 cm), or deep (26 cm) compost depths (Figure 1A,D), with the bottom layer labelled using ^15^N–NH_4_Cl. (A) Mushroom wet weight (ww) production per flush mean values + SE are shown. (B) Total yield expressed per kg of compost (wet weight). (C) Mean δ^15^N values (‰) ± SE of harvested mushrooms from flush 1 (F1, green bars) and flush 2 (F2, orange bars). (D) Mean δ^15^N values (‰) ± SE normalized for the total amount of ^15^N that was applied to the cultures. Letters denote Tukey HSD groupings. Differently coloured letters indicate comparisons either between flush 1 or flush 2.

Compost filling depth can be varied in cultivation to balance total yield with efficiency, defined as production per unit of compost. To test how compost bed depth affects the contribution of different substrate layers to feeding the mushrooms, we prepared box cultures at three depths: shallow (~16 cm), standard (~22 cm), and deep (~26 cm) (Figure 1A,D). The bottom layer of each box was labelled with ^15^N–NH_4_Cl at equal concentrations, and two flushes were harvested. As increasing bed depth also increased compost weight, production per box was also increased with increasing bed depth (Figure 6A). Yet, increased depth led to lower yields per kg compost as expected, but this was not significant (Figure 6B).

In the first flush, depth did not significantly affect ^15^N incorporation, though the trend suggested greater uptake from the bottom layer in shallower beds (Figure 6C). However, by the second flush, filling depth had a more clear effect. Deep beds mobilized significantly more ^15^N from the bottom layer than shallow beds (1825‰ ± 120 δ^15^N and 1562‰ ± 55 δ^15^N, respectively), with standard depth (control) showing intermediate uptake (Figure 6C). However, after normalization for the total ^15^N content present in the compost, the second flush on deep beds did not significantly contain more ^15^N anymore compared with the shallow beds (Figure 6D).

### 3.6 Nitrogen Translocation Within the Substrate

**FIGURE 7:**
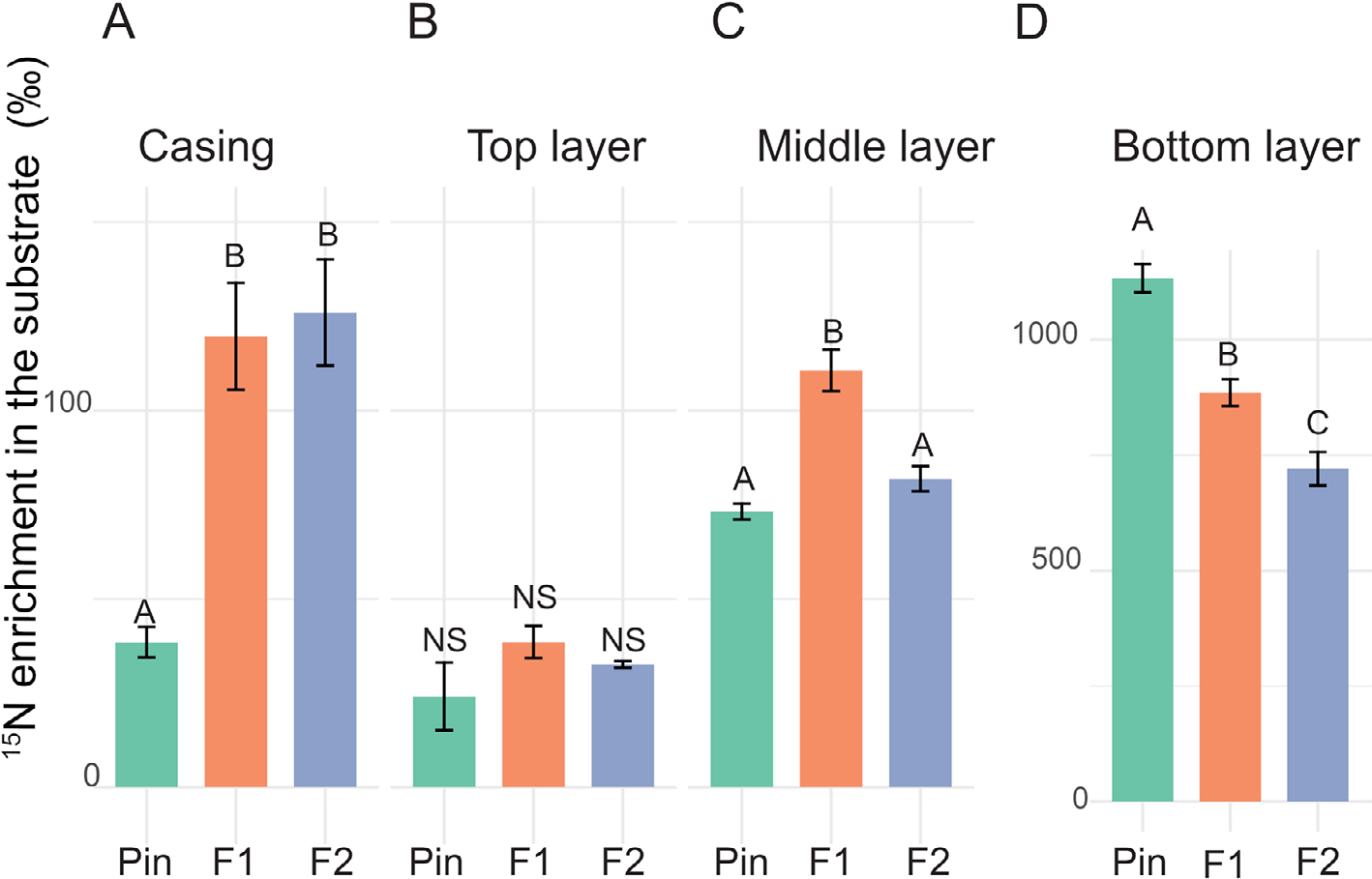
Translocation of ^15^N between bottom compost layer and other layers, measured before the first flush at Pinning (Pin), after the first flush (F1), and after the second flush (F2). (A) In the casing layer ^15^N increases during the first flush and remains steady. (B) In the top compost layer, no significant change in the amount of ^15^N is measured. (C) In the middle layer ^15^N increases during the first flush; this ^15^N is then used during the second flush. (D) Bottom layer ^15^N enrichment decreases during fructification as mushrooms take up ^15^N. ^15^N concentration in this layer is ~10× higher than in the other layers. Different letters denote significant differences between different cultivation stages (Tukey HSD); NS: Not significant (ANOVA).

To test whether nitrogen redistributes between compost layers, ^15^N was quantified in compost strata before flush one, after flush one, and after flush two in the control setup (Figure 1A). Overall, redistribution was limited. The middle layer showed a small enrichment after flush one (Figure 7C), suggesting some new growth occurred that temporarily incorporated ^15^N from the bottom layer. However, this effect was minor compared with the direct depletion of the bottom layer, which lost ^15^N progressively over both flushes (Figure 7D), due to transport to the mushrooms. The top layer showed no significant accumulation at any stage (Figure 7B). The casing layer behaved differently. Its dry weight increased by 4.5% ± 1.4%, and ^15^N rose to a steady level after flush one (Figure 7A), reflecting vegetative growth and pin formation.

### 3.7 Transport During Fruiting Body Development

**FIGURE 8:**
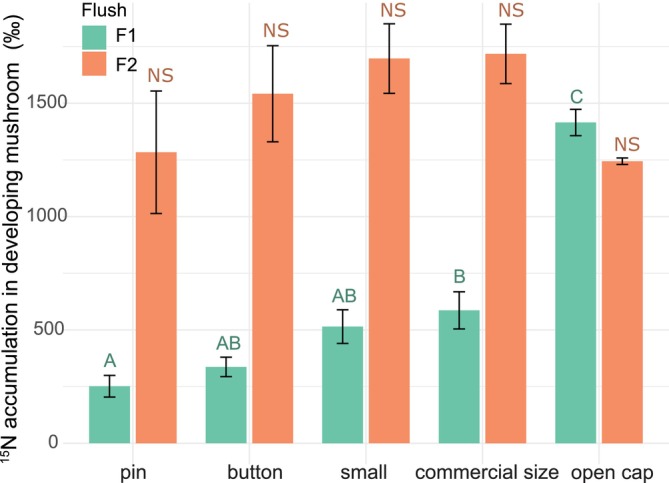
Bottom layer utilization quantified by ^15^N uptake at different stages of mushroom development shown as mean δ^15^N values (‰) ± SE. Different letters indicate significant differences between developmental stages during either the first or second flush indicated by different coloured letters (Tukey HSD); NS: Not significant (ANOVA).

To determine when *A. bisporus* begins translocating nitrogen over longer distances, we measured ^15^N here and previoulsy; protein and total nitrogen accumulation at successive developmental stages during two flushes. In flush 1, protein content and nitrogen concentration were highest in young pins and declined as mushrooms matured (van Iersel et al. 2025). This decline was paralleled by gradual increases in ^15^N derived from the bottom layer. A significant rise in ^15^N tracer uptake from the bottom layer occurred once mushrooms reached commercial picking size, coinciding with the peak flux of resources from substrate to fruiting bodies (Figure 8). During the second flush, ^15^N uptake from the bottom layer remained elevated across developmental stages and only declined again when mushrooms opened, but this effect was not significant.

## 4 Discussion

In this study we have shown that nutrient uptake and transport in *A. bisporus* are shaped by both mycelium architecture and colonization time. Our study shows how these factors interact, and how cultivation variables can alter the balance between local (top layers) and long‐distance resource mobilization (bottom layers). This is not only relevant for understanding nutrient transport, but also for improving biological efficiency and yield in mushroom production systems as the bottom layer remains underutilized in present cultivation systems (Sonnenberg et al. 2022; Vîta et al. 2026).

^15^N‐labelled ammonium was applied to the bottom compost layer to monitor the contribution of long‐distance nitrogen translocation from this region to developing mushrooms. However, the compost represents a complex microbial ecosystem in which bacteria are highly abundant and likely assimilate a substantial fraction of the added ammonium. As a result, labelled nitrogen reaching the fungus may partly originate from microbial biomass turnover (Kertesz and Thai 2018; Vos et al. 2017) rather than from direct ammonium uptake by *A. bisporus*. The tracer signal measured in mushrooms should therefore be interpreted as the integrated outcome of nitrogen mobilization from the labelled bottom layer, instead of exclusively direct uptake of the applied ammonium. Since all treatments were performed within the same compost matrix and microbial community, such microbial immobilization would likely affect treatments equally. The relative differences in tracer incorporation observed between treatments therefore still reflect differences in the contribution of nitrogen derived from the bottom compost layer to mushroom growth.

### 4.1 Cord Formation, Transport, and Compost Bed Depth

**FIGURE 9:**
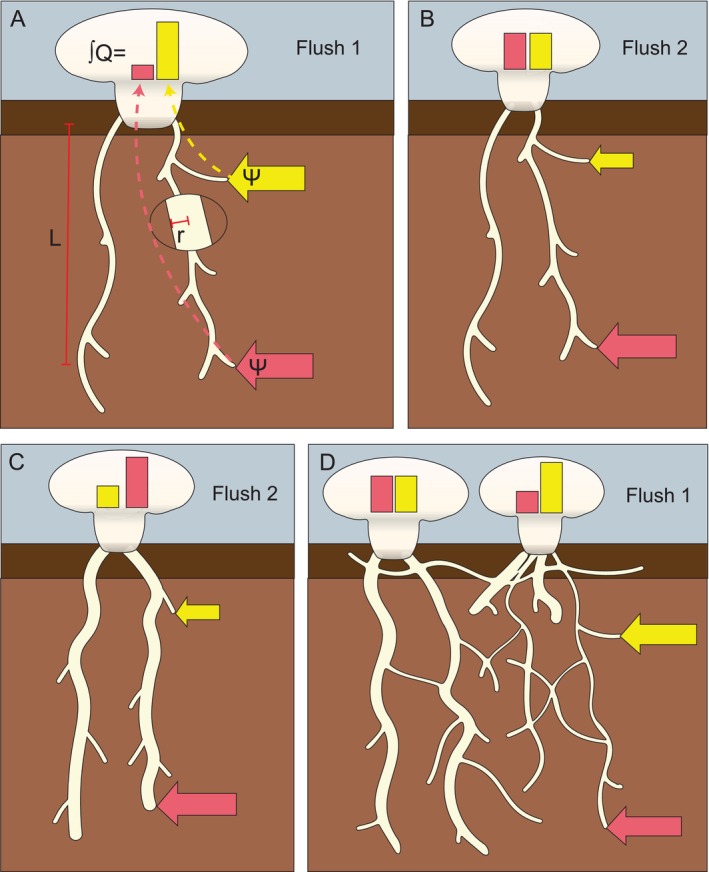
Conceptual model of resource uptake from different compost strata in *A. bisporus* cultivation. (A) Resource uptake from each stratum here defined as the cumulative flow to a growing mushroom (∫*Q*). Accumulated resources from different layers are represented by yellow (top layers) and red (bottom layers) bars. Resource flow is determined by the water potential (*∆Ψ*) generated by the mycelium in different layers and by network properties (distance *L*, hyphal radius *r*, and number of hyphae *n*) that together define the hydraulic resistance (Equation 2). At the start of cultivation, water potential is similar across strata, but hydraulic resistance of the network limits uptake from the bottom layer due to the longer distance to the mushrooms. (B) In flush 2, as top layers have been progressively depleted, the bottom layer maintains a stronger relative water potential, leading to increased resource utilization from larger depths. (C) When hydraulic resistance decreases for example, through the formation of wider or more abundant cords and upper layers become nutrient depleted, bottom‐layer utilization becomes more efficient. (D) Variation in connectivity to large cords linking different substrate layers likely explains heterogeneity in resource supply among individual mushrooms, even within a single interconnected mycelial network. The size of the arrows represents the strength of the water potential for top (yellow) and bottom layers (red). Cords are represented by wide filaments, whereas fine single hyphae are represented by filaments with low width.

Nutrient translocation in *A. bisporus* depends strongly on the distance over which resources must travel through the compost. Increasing the filling depth extends this distance and, according to the Hagen–Poiseuille law, increases hydraulic resistance and thereby limits the rate of water and nutrient flow through the mycelial system to the mushrooms. The total resource uptake by a given mushroom can be expressed as the time‐integrated flow (𝑉) towards that fruiting body over its period of development (Figure 9A), where *Q* is volume flow rate and *t* time.

(1) $$V\left(t\right)=\int_{0}^{t}Q\left(t^{’}\right)\;dt$$

In an interconnected network of hyphae and cords, several structural and physiological parameters govern this flow: the transport distance (𝐿), the number of hyphae or cords connecting to the fruiting body (𝑛), the effective hyphal radius (𝑟), the viscosity of the cytoplasmic fluid (𝜂), and the water potential gradient driving flow (*ΔΨ*). The volumetric flow rate through the system can be approximated by the Hagen–Poiseuille relation:

(2) $$Q=n\frac{\pi ∆{\mathrm{\Psi r}}^{4}}{8\mathrm{\eta L}}$$

Thus, the contribution of different compost layers or regions to mushroom growth (*V*) depends on the dynamic balance between hydraulic resistance, connectivity, and the local driving potential across the network. This theoretical framework can explain translocation using mass flow through the hypha. Here, we do not discuss other pathways of extracellular water translocation to the fruiting bodies as we previously found (Herman et al. 2025), which likely occur in tandem.

Surprisingly, we found that deeper filling depths did not significantly reduce bottom‐layer utilization from the bottom 1/3 of the substrate in the first flush, but did promote stronger mobilization of deeper resources in the second flush (Figure 6C). However, when normalizing for the total ^15^N present in the cultures, this effect was lost (Figure 6D). The former partially explains why deeper beds often yield more mushrooms despite lower efficiency per unit of compost (Sonnenberg et al. 2022). However, when increasing cultivation time up to 100 days, this biological efficiency decrease with increasing bed depth is not observed (Nielsen and Rasmussen 1962), which is in accordance with this theoretical framework. If cultivation is carried on for long enough (*t*, time), although economically unfeasible, the effect of hydraulic resistance becomes insignificant; then only the ability of the fungus to maintain a differential pressure potential determines its total growth.

Directional growth allows the fungus to establish a network with abundant cords running in the direction of transport, prior to fructification. Cords lower the resistance of extended transport pathways (Herman and Bleichrodt 2021), due to the presence of internal wide vessel hyphae (*r*, radius) and a large number of hyphae running in parallel (*n*, number of hyphae). During the first flush, we observed that this does not lead to a relative increase in bottom layer utilization (Figure 3B). Although counterintuitive, this makes sense with this theoretical framework (see also Figure 9A). Since the conditions in the substrate are still equal, the mycelium likely experiences the same internal pressure in all substrate layers. Moreover, resistance is reduced across the entire fungal network and not specifically from the deeper strata. Since flow rate is inversely proportional to pathway length (Equation 2), close by resources will be used first by the growing mushrooms. Therefore, the relative bottom layer use will not change with cord‐rich directional growth. However, the flow rate will increase; thereby, more nutrients will be transported from the substrate, resulting in higher yield (Figure 3A). By contrast, during the second flush, upper layers are depleted more, and mushrooms must resort to feeding from resources located within lower substrate layers. In this case, the transport distance increases (*L*), which increases the resistance to flow. Now, cords become even more relevant since they counteract this increased transport distance by lowering the resistance. Directional growth therefore increases long‐distance translocation during the second flush when the top compost layer has been relatively depleted more due to higher first flush yield (Figure 3B; Figure 9C).

Taken together, these results indicate that both physical distance and network architecture (cords) jointly determine to what extent substrate layers contribute to fruiting body development. Cords lower the resistance of extended transport pathways (Herman et al. 2020; Herman and Bleichrodt 2021). Directional growth therefore can increase yield in the first flush and increase long‐distance translocation during the second flush when the top compost layer has been depleted (Figure 9C). Unfortunately, directional growth is not economically feasible in a commercial setup since it would take an additional 3 weeks to colonize the compost before the casing layer will start to be colonized.

### 4.2 Individual Fruiting Body Heterogeneity

In most experiments we analysed pooled mushrooms, providing an average view of nutrient flow. However, measurements on individual fruiting bodies revealed that different mushrooms accessed nutrients from different substrate layers, as reflected in the variability in ^15^N tracer uptake (Figure 2). At first sight this is unexpected, given that the *A. bisporus* mycelium forms a highly interconnected network.

We considered whether competition between fruiting bodies or heterogeneous osmolyte accumulation could generate differential water potentials, but neither bottom‐layer utilization nor individual osmolyte profiles correlated with mushroom size (Tables S1 and S2) (see also van Iersel et al. 2025). Tracer redistribution between substrate layers was minimal (Figure 7), ruling out redistribution as an explanation for the observed heterogeneity, as mushroom growth was the main driver of translocation in the system.

This leads us to the hypothesis that the specific position of the fruiting body on the network and its connectivity and corresponding hydraulic resistance to specific substrate regions is the explanation for this heterogeneous feeding. Larger cords can have a lower resistance than smaller cords and fine hyphae (Fricker et al. 2017; Herman et al. 2020; Herman and Bleichrodt 2021), shaping the hydraulic pathways that dominate transport to specific mushrooms. A comparable pattern is seen in plants, where hydraulic sectoring causes anatomically continuous xylem networks to function as semi‐independent flow pathways (McElrone et al. 2021; Zanne et al. 2006). This underscores that physical connectivity alone does not ensure homogeneous transport.

Because transport follows pressure gradients with water and solutes moving from higher‐potential substrate zones towards sinks (e.g., fruiting bodies) (Heaton et al. 2012) the interplay of cord thickness, connectivity, and local water potentials will determine which substrate layer supplies a particular fruiting body to what extend. This provides a mechanistic explanation for why mushrooms in the same bed can draw resources from different substrate layers (Figure 2; Figure 9D). Based on the observed variance among individual mushrooms, pooling 10 mushrooms per box reduces the expected sampling error of the mean by ~√10 (SE = 142). Consequently, the variation introduced by heterogeneity among individual fruiting bodies is small relative to the observed differences reported in the other experiments.

### 4.3 Effect of Compost Age and Colonization Time

Directional growth introduces heterogeneity in mycelial age across the culture, which may influence nutrient dynamics independently of cord formation. To test this, we examined heterogeneous compost colonization time without directional growth and observed a different pattern. In this case, bottom layer ^15^N uptake increased significantly in the first flush (Figure 3A), showing that younger compost can already redistribute nutrients across the network to compensate for locally limited substrate breakdown, channelling resources from lower regions (Figure 9B). However, the first flush yield was much lower than with directional growth. This can be explained by the higher resistance to flow of the non‐directional network due to the low number of cords. In regards to the directional growth experiment, it seems that even though upper strata were only colonized for relatively short amounts of time (top layer 7 days colonized, middle layer 14 days colonized on average in directional growth, compared with 16 days colonization in non‐directional growth), the increased colonization density achieved through directional growth does not limit nutrient uptake from the upper strata during the first flush (Figure 3B). In heterogeneous compost in the second flush, this leads to a strong decline in bottom layer use, as the bottom layer was overly used in the first flush. Moreover, by that time the mycelium in the upper layer had more time to colonize and degrade the substrate. A similar pattern was observed when looking at casing water uptake, when less casing water was available. An increase in transport from the bottom compost layers was observed (Kalberer 1985). This highlights the fungus's innate ability to manage its resource acquisition and adapt to complex, changing environments.

Compost colonization time emerges as a factor influencing where resources are taken up from. In the earlier models of fungal nutrition (Chanter and Thornley 1978) it was proposed that the mycelium absorbs substrate at a constant rate and accumulates it until a threshold is reached, after which fruiting bodies develop and deplete the reserve. *A. bisporus* biomass approximation based on fungal Phospholipid fatty acid (PLFA) analysis of colonized compost shows that this is not the complete picture, an estimated 30.2 g dw living mycelium per kg compost (Vos et al. 2017), producing up to 58.8 g dw mushrooms per kg dw of compost during the first flush (Figures 4A and 5A). Thus, the dry matter stored in living mycelium on its own is insufficient to feed the developing mushrooms. Later work (Claydon et al. 1988) showed that extracellular enzyme activity fluctuates in synchrony with fruiting body biomass formation, indicating that nutrient acquisition is also demand‐driven and tightly coupled to the needs of developing mushrooms. This demand‐driven view aligns with the observation that efficient degradation of lignocellulosic biomass is essential for the conversion of compost into fruiting bodies (Iiyama et al. 1994; Jurak et al. 2015; Patyshakuliyeva et al. 2015; Vos et al. 2021). Substrate‐degrading enzymes are secreted prior to the first flush and, while relatively stable, they decrease in abundance during the second flush (Patyshakuliyeva et al. 2015).

We further tested the role of colonization time in a semi‐commercial trial with compost colonized for 14, 17, 20, or 30 days. When all layers had equal short colonization times, the mycelium did not have enough time to sufficiently degrade the substrate, indicated by the lower yield (Figure 4A). Therefore, we expected that the mushrooms would utilize all three layers for feeding to sustain the first flush. As expected, the bottom layer contributed less, as the mycelium experiences increased hydraulic resistance compared with the top layers (Figure 4B).

As expected, standard colonization times of 17–20 days produced stable yields. Surprisingly, very old compost (30 days) was the most productive, yielding more in the first flush. Shorter colonization times instead resulted in more balanced yields across the first two flushes (Figure 4A). This is surprising as substrate degradation also could have negative effects, particularly on water availability. As the substrate breaks down further, more osmotically active solutes such as mineral salts and trace metals are likely released from the substrate and accumulate. Simultaneously, water volume decreases due to uptake by the mushrooms. Together these processes may result in decreasing water potential, likely contributing to reduced productivity (Kalberer 1987). The shorter compost colonization times may also have affected colonization of the casing soil, but it was not our scope to disentangle the effects of compost and casing colonization separately on yield, but we assessed the system as a whole. Nevertheless, the timing of fruiting was almost similar for all treatments.

In contrast to the heterogeneous colonization time experiment (Figure 3B), the semi‐commercial trial did not reveal increased bottom‐layer use in younger compost during the first flush (Figure 4B). Although longer colonization time increased total resource availability, as reflected by mushroom yield (Figure 4A), the depth of uptake was similar across colonization times. However, during the second flush, compost colonization time had a significant effect. ^15^N uptake, corrected for depletion, increased strongly in all colonization times but most strongly in older compost (Figure 4B). This shows that older compost first exploits top layers more completely, then relies on bottom layers when upper resources are exhausted, thereby explaining the reduced second flush yield. Layer specific dry weight changes provided additional confirmation. Most dry matter was lost during the first flush (Figure 4C), but this includes respiration and nutrient transport feeding casing colonization as well as translocation to mushrooms. Still, relatively bottom‐layer dry weight declined less compared with the top and middle compost layers in the first flush and more in the second, mirroring the ^15^N data. Again, older compost tended to use more of the bottom layer in the second flush, although variability limited statistical power. Long‐distance translocation therefore is not impaired by extended colonization time, but instead appears conditional. It is engaged more when local resources are insufficient or depleted. This effect becomes more pronounced with increasing colonization time prior to the first flush.

Given that resource availability shifts between flushes, we next tested whether delaying the second flush would change how layers are exploited. Delaying the second flush would give the enzymes more time to further degrade the substrate, thereby providing more close by nutrients for feeding the mushrooms. Under standard conditions, bottom‐layer ^15^N uptake increased in the second flush as the fungus depended more strongly on more deeply located resources (Figure 5B). When we extended the interval between flushes by 11 days, 2^nd^ flush yields rose slightly, though not significantly (Figure 5A), but the usual increase in bottom‐layer contribution was absent. ^15^N uptake remained at first‐flush levels (Figure 5C). Thus, delaying fruiting decreased deeper resource mobilization while supporting high yields by maintaining a stronger reliance on upper layers. The total yield increase is similar to that seen in 30 days old compost. Only now the increase in yield is seen produced during the second flush. The extended inter‐flush interval likely allowed proximal hyphae to restore pressure generating capacity by increasing nutrient availability from substrate breakdown, so flow again preferentially followed the short, low‐resistance pathways in the upper compost, reducing the need to engage deeper strata. From our analysis of tracer movement across substrate layers in control boxes, it seems that redistribution of resources to depleted strata, if at all, is limited at the centimetre scale within the substrate layers (Figure 7). This is consistent with the smaller increase in mushroom nitrogen content observed during the second flush when its onset was delayed (van Iersel et al. 2025) (Figure 5B). These data further support that mushrooms grown on older compost are predominantly using the top layer in flush 1 (Figure 4B).

We next asked whether nutrient sourcing also changed over the course of fruiting body development. Mushrooms follow a consistent developmental sequence. Pins are initiated on the casing soil, and only a few continue to mature into fruiting bodies (Hammond and Nichols 1976; Straatsma et al. 2013). During this process, the composition of the fruiting body changes, with protein content decreasing as pins develop (van Iersel et al. 2025). We found no evidence that developmental stage itself determined the source of nutrient uptake. Instead, resource origin shifted once the bulk of transport had occurred. After mushrooms had expanded to commercial size, more nutrients were drawn from the bottom layer, likely because the top layers had become depleted (Figure 8). This shows that already within a flush, local nutrient availability can become limiting.

## 5 Conclusion

Our results show that nutrient translocation in *A. bisporus* is highly context‐dependent. Cords can facilitate long‐distance transport, but only when local resources are limiting in the upper substrate layer(s). Compost age, colonization time and depletion of certain zones determines whether nutrients are sourced locally or from deeper layers, while cultivation variables such as flush timing and filling depth further shape these dynamics. These findings highlight that long‐distance translocation is not a fixed property of the network, but a flexible response depending on resource distribution and network architecture. Increasing cord abundance across the network does not necessarily change resource uptake patterns, but can improve translocation efficiency by reducing hydraulic resistance across the network.

Our results showing the strong effects of colonization time on nutrient acquisition also connect to demand‐ and supply‐driven models of flush development (Claydon et al. 1988). Since our results resemble a supply‐driven pattern, the classic storage model (Chanter and Thornley 1978) is unlikely to hold as originally formulated. Instead, the “supply” component may reflect the fungus's ability to produce extracellular enzymes and the time allowed for the enzymes to work on the substrate. Thereby, these factors create access to nutrients when demand arises from developing mushrooms. This enables the uptake of nutrients and water, thereby creating a positive pressure driving mass flow towards the fruiting bodies.

For mushroom cultivation, this means that a longer colonization time of compost can increase the yield but will still lead to uneven utilization as bottom layers are only accessed after the top layer has been depleted. Decreasing hydraulic resistance across the compost by changing cultivation conditions (more cords or shallower beds) can increase flow rates across the network, making shorter cultivations more efficient. If compost prices rise, the employment of longer colonized compost and longer cultivation cycles may become economically feasible.

## Author Contributions

**Robert‐Jan Bleichrodt:** conceptualization, supervision, funding acquisition, writing – original draft, writing – review and editing. **Guus van Iersel:** conceptualization, methodology, investigation, formal analysis, writing – review and editing, writing – original draft. **Johan Baars:** investigation, writing – review and editing. **Pieter Vervoort:** investigation.

## Funding

This work was supported by Nederlandse Organisatie voor Wetenschappelijk Onderzoek, TTW Vidi grant ‘Feedme’ [18920].

## Conflicts of Interest

The authors declare no conflicts of interest.

## Supporting information


**Table S1:** Pairwise Pearson correlation coefficients (*R* ^2^), for 6 measured variables of 23 individual fruiting bodies originating from 3 independent cultivation boxes in flush 1 (Figure 1A).
**Table S2:**: Pairwise correlation *p*‐values, for 6 measured variables of 23 individual fruiting bodies originating from 3 independent cultivation boxes in flush 1.

## Data Availability

Raw data of this study is available at Figshare under https://doi.org/10.6084/m9.figshare.30818828.
