# Supplementary material for: Feeding of Agaricus bisporus Mushrooms is Impacted by Mycelium Colonization Time and Network Architecture
Source: Environ Microbiol. 2026 Jun 15;28(6):e70345. doi: 10.1111/1462-2920.70345 (PMC13268701; doi:10.1111/1462-2920.70345)
Supplement: Supplementary file 1 — Table S1: Pairwise Pearson correlation coefficients (R 2), for 6 measured variables of 23 individual fruiting bodies originating from 3 independent cultivation boxes in flush 1 (Figure 1A). Table S2:: Pairwise correlation p‐values, for 6 measured variables of 23 individual fruiting bodies originating from 3 independent cultivation boxes in flush 1. [file EMI-28-e70345-s001.docx]

**Supplementary Table 1**: Pairwise Pearson correlation coefficients (R^2^), for 6 measured variables of 23 individual fruiting bodies originating from 3 independent cultivation boxes in flush 1 (Fig. 1A).

| Pearson R2 | weight | δ^15^N | nitrogen % | amino acid % | protein % | osmotic pot. |
| --- | --- | --- | --- | --- | --- | --- |
| weight | 1 | 0.002 | 0.025 | 0.023 | 0.008 | 0.005 |
| δ^15^N | 0.002 | 1 | 0.327 | 0.057 | 0.141 | 0.111 |
| nitrogen % | 0.025 | 0.327 | 1 | 0.021 | 0.058 | 0.026 |
| amino acid % | 0.023 | 0.057 | 0.021 | 1 | 0.133 | 0.113 |
| protein % | 0.008 | 0.141 | 0.058 | 0.133 | 1 | 0.117 |
| osmotic pot. | 0.005 | 0.111 | 0.026 | 0.113 | 0.117 | 1 |

**Supplementary Table 2**: Pairwise correlation p-values, for 6 measured variables of 23 individual fruiting bodies originating from 3 independent cultivation boxes in flush 1.

| p-values | weight | δ^15^N | nitrogen % | amino acid % | protein % | osmotic pot. |
| --- | --- | --- | --- | --- | --- | --- |
| weight | 0 | 0.835 | 0.469 | 0.488 | 0.687 | 0.76 |
| δ^15^N | 0.835 | 0 | 0.004 | 0.272 | 0.078 | 0.121 |
| nitrogen % | 0.469 | 0.004 | 0 | 0.514 | 0.268 | 0.459 |
| amino acid % | 0.488 | 0.272 | 0.514 | 0 | 0.087 | 0.116 |
| protein % | 0.687 | 0.078 | 0.268 | 0.087 | 0 | 0.11 |
| osmotic pot. | 0.76 | 0.121 | 0.459 | 0.116 | 0.11 | 0 |
